# Case Report: Metachronous bilateral upper tract and bladder urothelial carcinoma: a long-term follow-up

**DOI:** 10.12688/f1000research.55516.2

**Published:** 2023-06-26

**Authors:** Agus Rizal Ardy Hariandy Hamid, Fakhri Zuhdian Nasher, Meilania Saraswati, Sahat Matondang, Chaidir Arif Mochtar

**Affiliations:** 1 Urology, University of Indonesia, Central Jakarta, Jakarta, 10430, Indonesia; 2 Anatomical Pathology, University of Indonesia, Central Jakarta, Jakarta, 10430, Indonesia; 3 Radiology, University of Indonesia, Central Jakarta, Jakarta, 10430, Indonesia; 4 Department of Urology, Cipto Mangunkusumo General Hospital, Faculty of Medicine Universitas, Indonesia, Jakarta, Indonesia

**Keywords:** Keywords: Upper Tract Urothelial Carcinoma, metachronous bilateral UTUC, Urothelial cancer recurrence, kidney sparing surgery, intracavity chemotherapy

## Abstract

**Background:** Upper tract urothelial carcinoma (UTUC) is a malignant disease of the urothelial cell lining the upper urinary tract from renal calyces, pelvises, and ureter down to the ureteral orifice. Urothelial carcinoma is a multifocal malignant tumor which tends to reoccur after treatment. Radical cystectomy shows that upper tract recurrence occurs in 0.75% to 6.4% of patients. The occurrence of contralateral UTUC after nephroureterectomy is rarer with a prevalence of 0.5%.

**Case presentation: **The case of a 43-year-old male with metachronous bilateral UTUC was reported. The patient had undergone gemcitabine-cysplatine neoadjuvant chemotherapy followed by radical cystectomy and orthotopic neobladder for urothelial carcinoma of the bladder cT2N0M0. Left hydronephrosis was discovered three months after the procedure. The patient was diagnosed with left UTUC cT4N0M0 of renal pyelum after a series of examinations. A left open radical nephroureterectomy was conducted to remove the mass followed by adjuvant chemotherapy. This was followed up with routine ultrasound and magnetic resonance imaging (MRI) every three months with a “tumor-free” period of 26 months. Meanwhile, the patient was re-admitted with fever and an increase in creatinine value of 4.3. After further workups, the patient was diagnosed with UTUC cT2N0M0 of the right renal pyelum. A kidney sparring approach with laser evaporation of the tumor was conducted followed by eight cycles of Gemcitabine intracavity antegrade per nephrostomy. After the regimen was finished, an MRI evaluation was conducted to assess treatment results, and the mass had decreased.

**Conclusions: **This report showed a rare case of urothelial cell carcinoma recurrences. From bladder urothelial carcinoma to left UTUC and then to contralateral UTUC. It is important to evaluate the upper tract to reduce the risk of recurrence.

## Introduction

Upper tract urothelial carcinoma (UTUC) is a malignant disease of the urothelial cell lining the upper urinary tract from the renal calyces or collecting system to the ureteral orifice. UTUC is considered a rare malignancy, representing 5% of urothelial cancer and less than 10% of all renal tumors ^1^ and it occurs 2-3 times more in males than females. ^2^ Urothelial carcinoma is a multifocal disease and tends to reoccur after initial treatment. The incidence of UTUC and collateral recurrency after the first tumor episode are also reported, and it is infrequent. ^3^^,^^4^ The overall prevalence after cystectomy ranges from 0.75% to 6.4%. Furthermore, the recurrence can appear at a range of 2.4 to 164 months. ^5^ The incidence of metachronous contralateral UTUC is also rare with a prevalence of 0.6%. This is manifested 9-71 months after the diagnosis of primary unilateral UTUC. ^4^ The occurrence of contralateral UTUC after nephroureterectomy is even rarer with the prevalence of 0.5% developing metachronous UTUC. ^4^ There are some risk factors like smoking and exposure to carcinogen contaminants in foods. ^6^ The metachronous contralateral recurrence also depends on some risk factors such as young age onset, small tumor size, and the history of bladder cancer. ^4^

This report aims to discuss a rare case of a patient at Ciptomangunkusumo National Hospital with a long history of urothelial carcinoma from the bladder. The patient had a recurrence in the left pyelum and after treatment, the right was affected.

## Case presentation

**Figure 1.**
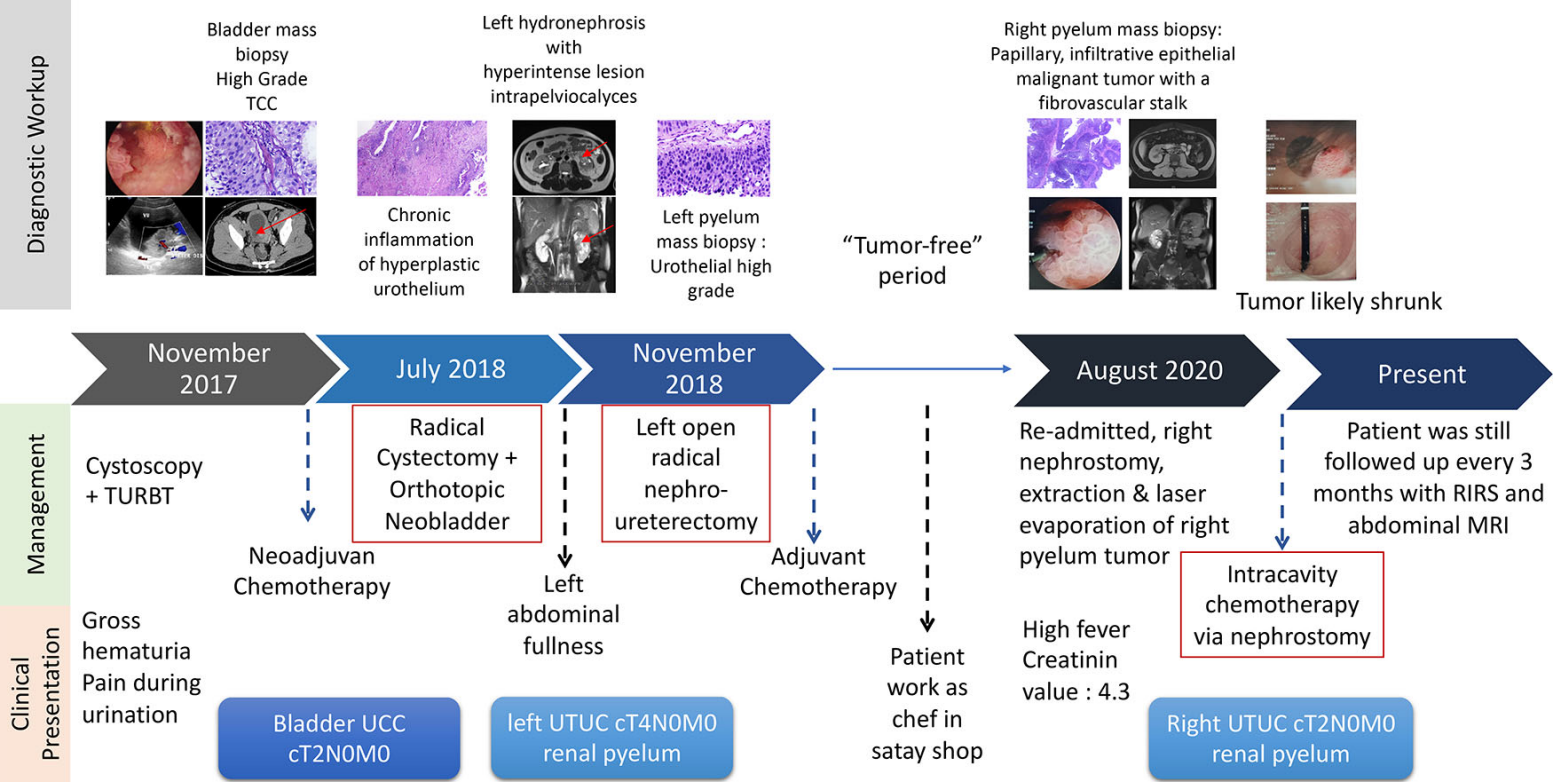
Timeline of the case report.

The patient is an Indonesian male born in 1978 who works as a cook. He was admitted to Ciptomangunkusumo National Hospital (RSCM) in November 2017 with gross haematuria as the chief complaint. The patient’s timeline is shown in Figure 1.

**Figure 2.**
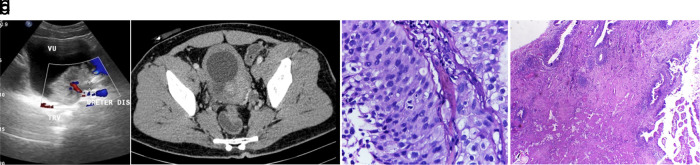
(a) Ultrasound in March 2020. An isoechoic lesion with irregular edges, on the left inferolateral bladder wall, and appears to be obstructing the left ureter with its distal part dilated. (b) Contrast computed tomography (CT) scan in December 2019 showing isodens mass that enhanced after contrast administration in the bladder. (c) The initial diagnosis was infiltrative high-grade urothelial carcinoma. The pictures show tumor cells with round/oval nuclei, pleomorphic, coarse chromatin, vesicular with nuclei, Hematoxylin and Eosin (H&E) stain 400×. (d) A follow-up biopsy showing urethra with minimal inflammatory infiltration. The picture showed that no tumor was found, chronic inflammatory, fibrinoid necrosis, H&E stain 100×.

The patient was an active smoker for 20 years and ate roasted meat regularly. From ultrasound and contrast computed tomography (CT) scan evaluation, a mass was discovered in the bladder ( Figure 2a). A cystoscopy and an incomplete transurethral resection of bladder tumour (TURBT) was then conducted on the papillary mass in the bladder. Furthermore, a biopsy was conducted, and pathology examination concluded that the tumor was an infiltrative papillar urothelial carcinoma pT1 high grade ( Figure 2b). In March 2018, the patient had bilateral hydronephrosis, and bilateral nephrostomy was confirmed. The bladder UCC was clinically diagnosed as cT2N0M1, and the patient was given 6-cycle neoadjuvant gemcitabine-cisplatin chemotherapy. During the chemotherapy phase, the patient was in good condition with a Karnofsky score of 90. In July 2018, a radical cystectomy followed by an orthotopic neobladder was conducted. Meanwhile, the frozen section of the right and left ureter, and urethral punctum showed no tumor. Pathology examination showed chronic inflammation of hyperplastic urothelium, with fibrinoid necrosis ( Figure 2c).

**Figure 3.**
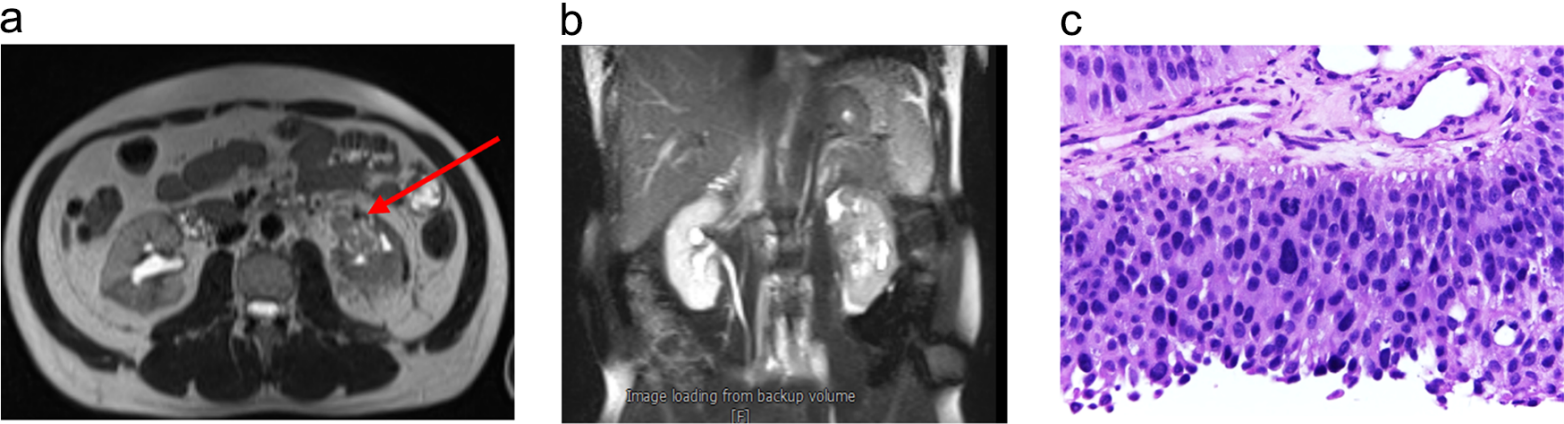
(a) Abdominal magnetic resonance imaging (MRI) with Gadobutrol 5 ml contrast, shown hyperintense lesions were seen on T1-T2WI and FS on the left intrapelviocalyceal and extracapsular perirenal, which were enhanced after the contrast was administered. (b) High-grade infiltrative urothelial carcinoma. (c) The picture shows papillary-arranged tumor mass, Hematoxylin and Eosin (H&E) stain 400×.

The patient was scheduled for adjuvant chemotherapy but was delayed due to the complaint of left abdominal fullness two months after the procedure. Ultrasound examination showed bilateral hydronephrosis with the left kidney being more severe. An abdominal MRI with contrast was performed ( Figure 3a). Furthermore, left nephrostomy and biopsy were conducted and pathology workup showed papillary arranged tumor mass. From diagnostic workup, the patient was diagnosed with left UTUC cT4N0M1 in renal pyelum ( Figure 3b).

**Figure 4.**
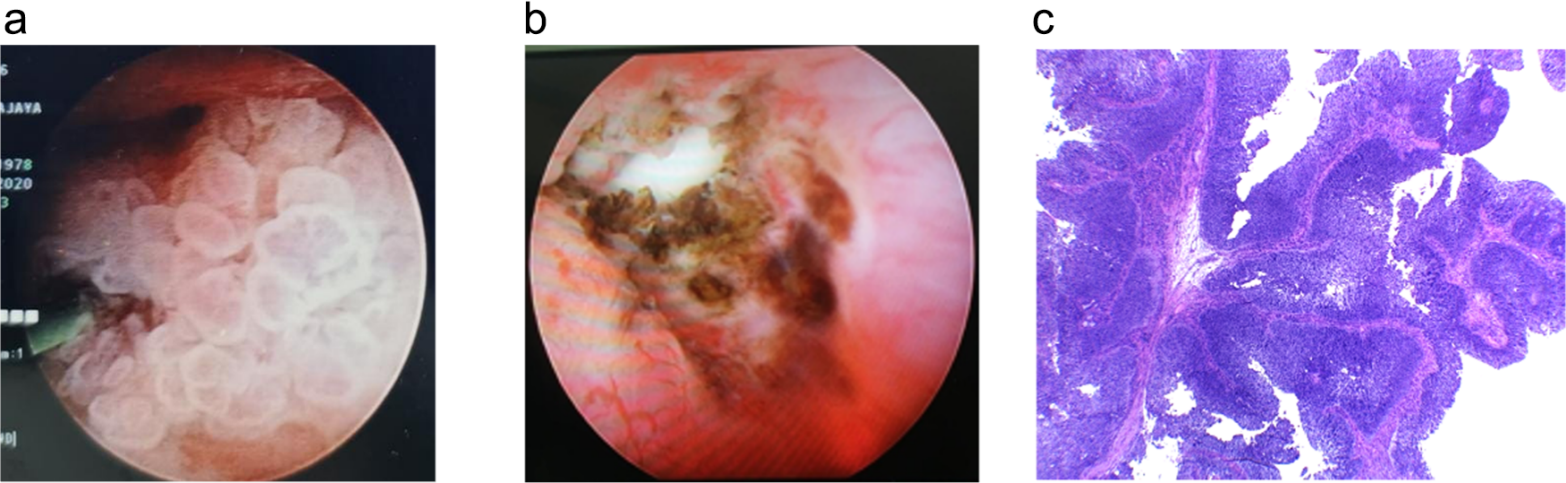
(a) Nephroscopy examination in pelviocalyceal system inserted through the right flank reveals a tumor sized about 3 cm. (b) Ureter post evaporation using laser shows some tumor residue even after laser procedure. (c) Tissue fragments containing papillary, infiltrative epithelial malignant tumors with fibrovascular stalk Hematoxylin and Eosin stain (H&E) stain 40×.

In November 2018, a left open radical nephroureterectomy was conducted to remove the mass, followed by six cycles of adjuvant chemotherapy of gemcitabine-cisplatin. Furthermore, a routine ultrasound and MRI were followed up every three months, and the indwelling catheter was replaced every two weeks. During the follow-up, the patient worked as a chef in satay shop and had a “tumor-free” period of 26 months with a Karnofsky score of 90. This continued until the patient was re-admitted with fever and an increase in creatinine value of 4.3 in August 2020. Then MRI was performed, and hyperintense solid mass was seen in the right renal pyelum. This mass measured 2.2 × 2.1 cm with left hydronephrosis grade II-III and surgery was planned for August 2020. In addition, a puncture was made in the right flank to the superior calyx guided by fluoroscopy into the pelvicalyceal system during the surgery. A nephroscope was then inserted, which exposed a tumor on the right pyelum sized about 3 cm ( Figure 4a). The tumor was then extracted using forceps for biopsy, and the residue was evaporated using a laser. Furthermore, nephroscope post-laser ablation examination shows some tumor residue ( Figure 4b). The biopsy result was a urothelial carcinoma infiltrative high grade.

**Figure 5.**
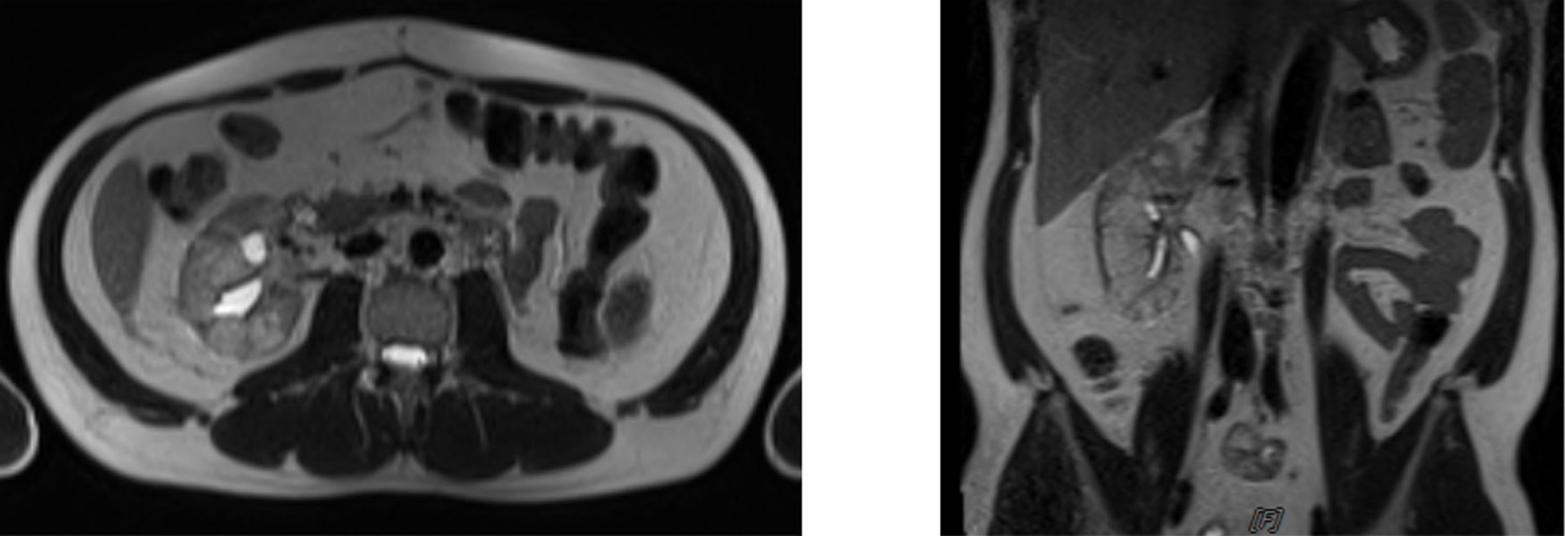
Abdominal magnetic resonance imaging (MRI) May 2021 shows benign calyx dilatation suspicious due to stricture, heterogeneous renal parenchyma.

The patient was given intracavitary chemotherapy using gemcitabine through nephrostomy. Furthermore, cystoscopy intracavitary instillation of chemotherapy agent using gemcitabine was administered through nephrostomy. The examination conducted in September 2020 showed a normal right kidney with no hydronephrosis, but there was a sign of intraluminal neobladder clotting. The patient then went to a clot evacuation cystoscope, and the last cycle of intracavitary gemcitabine was given in November 2020. Furthermore, an MRI and ureterorenoscopy were conducted and the mass was shown to be decreased. Abdominal MRI in May 2021 showed benign calyx dilatation suspicious due to stricture and heterogeneous renal parenchyma ( Figure 5).

## Discussion

Upper tract urothelial carcinoma (UTUC) is a malignant disease of the urothelial cell lining the upper urinary tract from the renal calyces or collecting system to the ureteral orrifice. UTUC is considered a rare malignancy, representing 5% of urothelial cancer and less than 10% of all renal tumors. ^1^ and it occurs 2-3 times more in males than females. ^2^ Generally, cancer has some risk factors that are categorized into endogenous and exogenous. Some endogenous factors are non-modifiable such as biological aging and genetic susceptibility while others are partially modifiable such as inflammation and hormones. ^6^ The example of genetic susceptibility is demonstrated in a meta-analysis by Chen, et al. (2016), where the correlation between HER2 expression and prognosis of UTUC was analyzed. This study showed that HER2 expression is significantly associated with a higher stage of tumor and worse recurrence-free in UTUC patients. ^7^ The exogenous factors are the most modifiable and are often modified to prevent cancer in the long term. These factors include radiation coming from radiologic examinations, chemical carcinogens from burnt food, and lifestyles including smoking, obesity, and lack of exercise. ^6^ Furthermore, urothelial carcinoma is a multifocal disease that may reoccur after initial treatment. This tendency can make a second tumor arise in another site with the urothelial cell as its lining, including the bladder.

In November 2017, the patient was admitted to Ciptomangunkusumo National Hospital with a chief complaint of gross hematuria, which is a common symptom of bladder tumors. Ultrasound, CT, and pathology confirmed that the patient had a urothelial cell carcinoma of the bladder and in July 2018, a radical cystectomy with an orthotopic neobladder was conducted. The patient had a left abdominal fullness and intrapyelum mass three months after radical cystectomy. With further workup, the patient was diagnosed with UTUC cT4N0M1 of the left pyelum. Furthermore, the odd of this recurrency is rare, especially after a radical cystectomy. A meta-analysis of 27 studies showed that the incidence of UTUC is rare and ranged from 0.75% to 6.4%. This was reported to occur as early as 2.4 to 164 months after cystectomy. ^5^

The patient is an active smoker and works as a cook in a satay shop. This exposed the patient to smoke and admitted eating roasted meat frequently. Smoking in particular is associated with advanced-stage disease, recurrence, and cancer-specific mortality in a patient treated with radical nephrouretectomy (RNU) for UTUC. ^8^ Furthermore, this also includes inhalation of smoke fume from cooking. ^9^ Cooking fumes are known to contain several mutagens such as 2-naphtylamine and 4-aminobiphenyl that can cause UTUC, ^9^ and consumption of processed meat also increases the occurrence of UTUC. ^10^ In addition, several meat preparations methods such as stewing and roasting can increase the risk of UTUC. ^10^^,^^11^ For high-risk UTUC, open RNU with bladder cuff excision is the standard treatment. This can be performed either open or with a laparoscopic approach. Lymph node dissection is also recommended to reduce the risk of local occurrence. ^12^

The patient was then declared cancer-free for 26 months before returning with fever and an increase in creatinine value of 4.3 in August 2020. Furthermore, abdominal MRI and anterograde evaluation using flexible URS (Ureterorenoscopy) and pathology report confirmed the diagnosis of right UTUC cT2N0M0 of renal pyelum. The biopsy resulted in a high-grade infiltrative urothelial carcinoma, and because of the history of previous radical nephroureterectomy of the left kidney, the sparring strategies were implemented on the patient. The incidence of metachronous contralateral UTUC was rare and according to a cohort study that follows up 23.075 patients with unilateral UTUC, only 144 (0.6%) developed metachronous UTUC. This was manifested after 9-71 months after the diagnosis of primary unilateral UTUC. ^4^ The occurrence of contralateral UTUC after nephroureterectomy was even rarer, and from the 12.382 patients with unilateral UTUC treated with nephroureterectomy, only 63 (0.5%) developed metachronous UTUC. ^4^ This development was not associated with survival outcomes of a patient with UTUC regardless of tumor stage. ^4^

There are some risk factors in determining the occurrence of metachronous contralateral UTUC. The same study that states the rarity of metachronous contralateral UTUC showed that younger age and smaller tumor size increase the risk of contralateral recurrence. Furthermore, the history of bladder cancer is also an important risk factor. ^4^ The onset is in the younger age range, and the tumor size, which is only 3 cm, and also has a history of bladder cancer and radical cystectomy. The patient remained a cook and was continuously exposed to smoke during the 26 months cancer-free period as well as frequently eating roasted meat, which contributed to the recurrence of UTUC metachronously.

According to the EAU (European Association of Urology) guideline for risk stratification, the patient experienced hydronephrosis, had a history of recurrency, and a biopsy showing high-grade infiltrative properties, so the second recurrence was categorized as a high-grade UTUC. ^12^ To the best of our knowledge, no case in the literature discussing the same clinical setting of UTUC recurrences in three different places especially after previous radical cystectomy and nephroureterectomy has been reported. It was then decided that the patient should undergo gemcitabine intracavity instillation eight cycle regiment. This gemcitabine regimen is analog to BCG (Bacillus Calmette–Guérin) or Mitomycin C regiment used intracavitary in low-risk UTUC guideline. ^12^ In addition, the installation of chemotherapy can be conducted through nephrostomy or retrograde through a single J open-ended ureteric stent. These regimens can be used as adjuvant therapy to decrease recurrent rate or as first-line therapy. ^12^ BCG instillation is widely reported in several case reports, but it is only used for low-risk carcinoma such as carcinoma *in situ* UTUC. A study using BCG instillation for first-line treatment of UTUC carcinoma in situ showed a 90% complete recovery rate. ^13^ Furthermore, a meta-analysis showed that both anterograde and retrograde have the same rate of complete recovery, overall survival, and recurrent rate. A retrospective study of 58 patients with UTUC carcinoma *in situ* also compares BCG instillation and radical nephroureterectomy as a treatment of choice. There was no difference in progression-free and overall survival between the two groups. ^14^ This is because both BCG and Mitomycin C are not available and, gemcitabine was used instead for a intracavity instillation regimen. A randomized control trial conducted at 23 US centers showed that Gemcitabine therapy decreases four years of recurrent risk from 47% with placebo to 35% in patients with non-muscle invasive urothelial cancer. ^15^ Also, a meta-analysis from 386 subjects and five pooled trials showed that as adjuvant therapy, there were no statistical differences in risk of recurrence and progression in a patient with non-muscle invasive bladder cancer. ^16^ The two studies showed a potential for gemcitabine to replace BCG as an intravesical chemotherapy agent. Histologically, both UTUC and urothelial carcinoma of the bladder consists of a neoplastic urothelial cell. The application of gemcitabine to replace BCG for topical installation can also be applied for UTUC. Meanwhile, no previous study has explained or reported the use of gemcitabine for intracavity installation through nephrostomy.

In this patient, gemcitabine 8 cycle was applied per antegrade through nephrostomy until November 2020. In addition, MRI evaluation of the post-gemcitabine intracavity regimen showed a decrease of the mass in the right pyelum. Kidney sparring treatment using topical intracavity chemotherapy after laser evaporation is still adequate to clear the tumor even at high-risk UTUK. The surgery is associated with shorter 5-years and 10-years overall survival in grade 3 UTUC. ^17^ Kidney sparring is associated with shorter 5-years and 10-years local recurrence-free survival even though there are no significant differences in 5-year metastasis with RNU. Furthermore, it is associated with an increased risk of upstaging compared to RNU which is particularly visible for grade 2 and 3 UTUC. ^17^

## Conclusion

This study reported a rare case of urothelial cell carcinoma recurrences. This was manifested in the bladder and left kidney pyelum treated with radical cystectomy and nephroureterectomy respectively. Also, another recurrence was reported contralateral in the right kidney pyelum. Therefore, genetic and risk factor exploration should be considered in young urothelial bladder cancer. This is important to evaluate upper tract in bladder urothelial carcinoma and reduce the risk of recurrency.

## Data availability

All data underlying the results are available as part of the article and no additional source data are required.

## Consent

Written informed consent for publication of their clinical details and clinical images was obtained from the patient and the family of the patient.
