# Supplementary material for: Case Report: Metachronous bilateral upper tract and bladder urothelial carcinoma: a long-term follow-up
Source: F1000Res. 2023 Jun 26;10:778. Originally published 2021 Aug 9. [Version 2] doi: 10.12688/f1000research.55516.2 (PMC11826075; doi:10.12688/f1000research.55516.2)
Supplement: Peer review history

# Reviewer report: Reviewer response for version 2

**DOI:** 10.5256/f1000research.151899.r363533

**Published:** 2025-02-13

**Authors:** Katsuhiro Ito

**Affiliations:** 1 Department of Urology, Ijinkai Takeda General Hospital, Ishida Fushimi-ku, Kyoto, Japan

This is a case report of a patient who experienced sequential bladder, right, and left urothelial carcinoma. I think it is worth sharing this case, although it is not a super rare case and we sometimes encounter such cases. Before ****indexing **,** extensive revision is necessary.

 Specific points:

 1. I was confused about the staging.

 "The bladder UCC was clinically diagnosed as cT2N0M1" Is this a metastatic case?

 2. Figure legends and text doesn't match.

 "tumor was an infiltrative papillar urothelial carcinoma pT1 high grade ( Figure 2b). " Figure 2b is a CT image.

 3. Is the biopsy picture shown in Figure 2D at the time of surgery or follow-up? I was confused.

 4. What was the pathological diagnosis of bladder cancer after neoadjuvant treatment? Please describe whether the tumor remained or completely regressed.

 5. I was surprised at the quick recurrence of the left kidney lesion. Is there any sign (CT image) of left kidney mass before cystectomy?

 6. I was also confused about the staging. 

 "the patient was diagnosed with left UTUC cT4N0M1 in renal pyelum" Does this patient have a metastatic lesion?

 7. Pathological staging of the left pelvic tumor should be described. 

 8. "the indwelling catheter was replaced every two weeks." What does this sentence mean? Did the patient have a nephrostomy until the recurrence?

 9. Please provide the result if you took a urine cytology test during the disease course. 

 10. Throughout the manuscript, reference numbers are not correct. Please check it again.

 "According to the EAU (European Association of Urology) guideline for risk stratification, the patient experienced hydronephrosis, had a history of recurrence, and a biopsy showing high-grade infiltrative properties, so the second recurrence was categorized as a high-grade UTUC. ^12^" I didn't check all but this #12 should be #6. 

 11. In conclusion, the authors stated that "Therefore, genetic and risk factor exploration should be considered in young urothelial bladder cancer." Is there any genetic background on this patient (e.g. Lynch syndrome)? No discussion was made on this point.

 12. " **This** is important to evaluate upper tract in bladder urothelial carcinoma and reduce the risk of recurrency." This sentence is unclear to me. What is " **this**"?

 We routinely check upper tract recurrence after cystectomy. Why is it particularly important in the case presented? Did this patient skip the follow-up test of the upper tract?

Are enough details provided of any physical examination and diagnostic tests, treatment given and outcomes?

Partly

Is the case presented with sufficient detail to be useful for other practitioners?

Partly

Is sufficient discussion included of the importance of the findings and their relevance to future understanding of disease processes, diagnosis or treatment?

Partly

Is the background of the case’s history and progression described in sufficient detail?

Yes

Reviewer Expertise:

Urology, physician.

I confirm that I have read this submission and believe that I have an appropriate level of expertise to confirm that it is of an acceptable scientific standard, however I have significant reservations, as outlined above.

# Reviewer report: Reviewer response for version 1

**DOI:** 10.5256/f1000research.59097.r129088

**Published:** 2022-04-12

**Authors:** Mohamed Mohamed Elawdy

**Affiliations:** 1 Urology Department, Sohar Hospital, Ministry of Health, Sohar, Oman

Thank you for the invitation to review this article. My notes are as follows:

 "Radical cystectomy shows that upper tract recurrence occurs in 0.75% to 6.4% of patients." Please elaborate on this sentence.

 "Upper tract urothelial carcinoma (UTUC) is a malignant disease of the urothelial cell lining the upper urinary tract from renal calyces, pelvises, and ureter down to ureteral orifice." This is a complicated sentence, instead perhaps you can write: "from the renal calyces or collecting system to the ureteral orifice."

 If the case is a UTUC after bladder cancer, why did you deal with contralateral UTUC after nephroureterectomy to such a degree?

 If you mention a case with multiple events, it is best practice to start with the most recent, then to mention the past history. Additionally, there is too much focus on the details of the patient’s habits.

 "An abdominal MRI with contrast was performed (Figure 3a). Furthermore, left nephrostomy and biopsy were conducted and pathology workup showed papillary arranged tumor mass." Why left nephrostomy for a biopsy? Why you did not get it with URS?

 Figure 2. "(a) Contrast computed tomography (CT) scan in December 2019 showing isodens mass that enhanced after contrast administration in the bladder. (b) Ultrasound in March 2020. An isoechoic lesion with irregular edges, on the left inferolateral bladder wall, and appears to be obstructing the left ureter with its distal part dilated." Please correct, figure (a) is the ultrasound.

 Figure 3. "(a) Abdominal magnetic resonance imaging (MRI) with Gadobutrol 5 ml contrast, shown hyper- intense lesions were seen on T1-T2WI and FS on the left intrapelviocalyceal and extracapsular perirenal, which were enhanced after the contrast was administered. (b) High-grade infiltrative urothelial carcinoma." Please correct, figure ( b) is the MRI.

 Furthermore, a routine ultrasound and MRI were followed up every three months, and the indwelling catheter was replaced every two weeks." Imaging is every 6 months, so why it was done every 3 months? What do you mean by indwelling catheter was replaced every two weeks?

 What do you mean by Karnofsky score of 90?

 "...right renal pyelum" This should be written as renal pelvis.

 "In addition, a puncture was made in the right flank to the superior calyx guided by fluoroscopy into the pelvicalyceal system during the surgery. A nephroscope was then inserted, which exposed a tumor on the right pyelum sized about 3 cm." Taking a biopsy by puncturing the kidney is not a standard. Why was this done? You can give a rational saying being a tumor in solitary kidney or so.

 "Furthermore, nephroscope post-laser ablation examination shows some tumor residue (Figure 4b). The biopsy result was a urothelial carcinoma infiltrative high grade." Can you mention the pathology clearly, was it non-muscle or muscle invasive?

 Overall, the discussion is very long for a case report, please shorten as much as possible.

 You should generally have a message from the case report, and the only massage is treating UTUC in a single kidney with resection and BCG. This is the rationale for this case to be accepted. Focus on this point and bring similar studies and make the discussion mainly on this point.

Are enough details provided of any physical examination and diagnostic tests, treatment given and outcomes?

Yes

Is the case presented with sufficient detail to be useful for other practitioners?

Partly

Is sufficient discussion included of the importance of the findings and their relevance to future understanding of disease processes, diagnosis or treatment?

Yes

Is the background of the case’s history and progression described in sufficient detail?

Yes

Reviewer Expertise:

NA

I confirm that I have read this submission and believe that I have an appropriate level of expertise to confirm that it is of an acceptable scientific standard, however I have significant reservations, as outlined above.

# Response

**Published:** 2022-11-10

**Authors:** Agus Rizal Ardy Hariandy Hamid

**Affiliations:** Indonesian Association of Urologists, Indonesia

Thank you for the comments, the following are our responses to the review:

- "Radical cystectomy shows that upper tract recurrence occurs in 0.75% to 6.4% of patients." Please elaborate on this sentence.

Upper urinary tract disease may develop in the residual transitional tissue in patients who have radical cystectomy for urothelial carcinoma. The odd of this recurrence is rare, especially after a radical cystectomy. A meta-analysis of 27 studies showed that the incidence of UTUC ranged from 0.75% to 6.4%. Numerous risk factors for upper urinary tract recurrence have been found in previous studies, however, it is still debatable how well each one predicts future occurrences.

- "Upper tract urothelial carcinoma (UTUC) is a malignant disease of the urothelial cell lining the upper urinary tract from renal calyces, pelvises, and ureter down to ureteral orifice." This is a complicated sentence, instead perhaps you can write: "from the renal calyces or collecting system to the ureteral orifice."

Thank you for your constructive feedback. Yes, we will paraphrase to: “Upper tract urothelial carcinoma (UTUC) is a malignant disease of the urothelial cell lining the upper urinary tract from the renal calyces or collecting system to the ureteral orifice."

- If the case is a UTUC after bladder cancer, why did you deal with contralateral UTUC after nephroureterectomy to such a degree?

This is the reason why we present this rare case of multiple event recurrence of UTUC which possible to make a second tumor arise in another site with the urothelial cell as its lining

- If you mention a case with multiple events, it is best practice to start with the most recent, then to mention the past history. Additionally, there is too much focus on the details of the patient’s habits.

Well noted, thank you for the feedback

- "An abdominal MRI with contrast was performed (Figure 3a). Furthermore, left nephrostomy and biopsy were conducted and pathology workup showed papillary arranged tumor mass." Why left nephrostomy for a biopsy? Why did you not get it with URS?

Thank you for your question, we would like to clarify that the nephrostomy mentioned in the line above was related to the management of the hydronephrosis of the left kidney that is more severe. The biopsy itself was done via URS

- Figure 2. "(a) Contrast computed tomography (CT) scan in December 2019 showing isodens mass that enhanced after contrast administration in the bladder. (b) Ultrasound in March 2020. An isoechoic lesion with irregular edges, on the left inferolateral bladder wall, and appears to be obstructing the left ureter with its distal part dilated." Please correct, figure (a) is the ultrasound.

Well noted, thank you for the correction, we will swap the picture in Figure 2

- Figure 3. "(a) Abdominal magnetic resonance imaging (MRI) with Gadobutrol 5 ml contrast, shown hyper- intense lesions were seen on T1-T2WI and FS on the left intrapelviocalyceal and extracapsular perirenal, which were enhanced after the contrast was administered. (b) High-grade infiltrative urothelial carcinoma." Please correct, figure (b) is the MRI.

Both Figure 3 (a) and (b) are the Abdominal MRI with different planes (axial and coronal view)

- Furthermore, a routine ultrasound and MRI were followed up every three months, and the indwelling catheter was replaced every two weeks." Imaging is every 6 months, so why it was done every 3 months? What do you mean by indwelling catheter was replaced every two weeks?

After a cystectomy, some patients who are at a higher risk of recurrence might benefit from stricter upper tract surveillance. Early diagnosis of recurrent disease allows for prompt administration of the proper therapy and may even enhance patient outcomes. Regular computerized tomography with urography enables the investigation of the upper urinary system from an oncological and functional perspective as well as the detection of any recurrence. (1) The indwelling catheter is regularly changed to reduce the risk of Ca-UTI. The timing of catheter changes should be individualized.

 (1) Picozzi S, Ricci C, Gaeta M, Ratti D, Macchi A, Casellato S, Bozzini G, Carmignani L. Upper urinary tract recurrence following radical cystectomy for bladder cancer: a meta-analysis on 13,185 patients. The Journal of urology. 2012 Dec 1;188(6):2046-54.

- What do you mean by Karnofsky score of 90?

According to Crooks et al., The Karnofsky Performance Scale Index allows patients to be classified as to their functional impairment. This can be used to compare the effectiveness of different therapies and to assess the prognosis in individual patients. In this setting, Karnofsky Score 90 means the patient is still able to carry on the normal activity with minor signs or symptoms of the disease. (Crooks, V, Waller S, et al. The use of the Karnofsky Performance Scale in determining outcomes and risk in geriatric outpatients. J Gerontol. 1991; 46: M139-M144)

- "...right renal pyelum" This should be written as renal pelvis.

Noted, thank you for the correction

- "In addition, a puncture was made in the right flank to the superior calyx guided by fluoroscopy into the pelvicalyceal system during the surgery. A nephroscope was then inserted, which exposed a tumor on the right pyelum sized about 3 cm." Taking a biopsy by puncturing the kidney is not a standard. Why was this done? You can give a rational saying being a tumor in solitary kidney or so.

Thank you for your comment, many recommend the ureteroscopic approach in the case of tumor biopsy. In the case a tumor in solitary kidney or a large tumors that would require long operatory time or even multiple procedures for a complete treatment, the antegrade approach is preferred. In this case, the tumor was then extracted using forceps for biopsy, and the residue was evaporated using a laser.

- "Furthermore, nephroscope post-laser ablation examination shows some tumor residue (Figure 4b). The biopsy result was a urothelial carcinoma infiltrative high grade." Can you mention the pathology clearly, was it non-muscle or muscle invasive?

Muscle invasive

- Overall, the discussion is very long for a case report, please shorten as much as possible.

Well noted, thank you for your kind feedback

- You should generally have a message from the case report, and the only massage is treating UTUC in a single kidney with resection and BCG. This is the rationale for this case to be accepted. Focus on this point and bring similar studies and make the discussion mainly on this point.

Thank you for your constructive feedback related to our article/manuscript

# Reviewer report: Reviewer response for version 1

**DOI:** 10.5256/f1000research.59097.r95637

**Published:** 2021-11-04

**Authors:** Alcides Chaux

**Affiliations:** 1 Department of Scientific Research, Norte University, Asunción, Paraguay

In this article, the authors report the clinicopathologic and outcome features of a patient with metachronous bilateral upper tract urothelial carcinoma. Overall, the case is well presented and the authors provide an appropriate background for their findings. A few recommendations are provided below.

- In the text, please provide the patient’s age instead of his year of birth.
- In Figure 1, please provide a consistent nomenclature for the histopathologic diagnoses. In all cases, the diagnosis should be “invasive urothelial carcinoma”. The use of descriptive terms such as “urothelial high grade” or “papillary, infiltrative epithelial malignant tumor with a fibrovascular stalk” could be confusing. The term “TCC” is also inappropriate. From the microphotographs presented by the authors, the tumor seems to correspond to the usual variant of invasive urothelial carcinoma, so no further specification is required.
- In Figure 1 and in the text, UCC should be UC. I would argue against using UC at all, recommending spelling “urothelial carcinoma” out at all instances to render the text clearer. 
- In the text, the description of Figure 2c does not correspond to the actual microphotograph. Figure 2d is not cited in the text. Please revise.
- Figures 3c and 4c are not cited in the text. Please revise.
- A few typos that need correction (reocurr, UTUK).

Are enough details provided of any physical examination and diagnostic tests, treatment given and outcomes?

Yes

Is the case presented with sufficient detail to be useful for other practitioners?

Yes

Is sufficient discussion included of the importance of the findings and their relevance to future understanding of disease processes, diagnosis or treatment?

Yes

Is the background of the case’s history and progression described in sufficient detail?

Yes

Reviewer Expertise:

Pathology, genitourinary

I confirm that I have read this submission and believe that I have an appropriate level of expertise to confirm that it is of an acceptable scientific standard.
